# Seasonal variation of pesticides in surface water and drinking water wells in the annual cycle in western Poland, and potential health risk assessment

**DOI:** 10.1038/s41598-022-07385-z

**Published:** 2022-02-28

**Authors:** Roksana Kruć-Fijałkowska, Krzysztof Dragon, Dariusz Drożdżyński, Józef Górski

**Affiliations:** 1 Department of Hydrogeology and Water Protection, Institute of Geology, Adam Mickiewicz University in Poznań, Bogumiła Krygowskiego 12, 61‑680 Poznań, Poland; 2 Department of Pesticide Residue Research, Institute of Plant Protection-National Research Institute, Władysława Węgorka 20, 60‑318 Poznań, Poland

**Keywords:** Environmental monitoring, Environmental sciences, Environmental impact, Hydrology

## Abstract

Drinking water wells on a riverbank filtration sites are exposed to contamination from farmlands (like pesticides) that had migrated from the contaminated river. In this study, pesticide contamination of the Warta River and riverbank filtration water at the Mosina-Krajkowo well field (Poland) were examined during the annual cycle. Among the 164 pesticides analysed, 25 were identified. The highest concentrations occurred in the river water and decreased along the flow path from the river to wells. Only the most persistent substances were detected at the farthest points. During the study, seasonal changes in pesticide concentrations and differences in the types of occurring substances were observed. Most substances and the highest concentrations were detected in May 2018, while the lowest number and the lowest concentrations were detected in February 2018. Spring is the period of increased exposure of water to pollution, which is correlated with increased pesticides use and increased rainfall. Seven toxic and persistent pesticides were found with the highest concentrations in water: isoproturon, nicosulfuron, imidacloprid, terbuthylazine, chlorotoluron, S-metalachlor, and prometryn. Pesticides are widely used in the study area; therefore, a potential health risk assessment was performed. The hazard quotient (HQ) values did not exceed one, which indicated a less significant health risk.

## Introduction

Many benefits in terms of the enhanced quality and quantity of crop production cause the use of pesticides in agriculture^1–3^. Pesticides are used to control insects, fungi, bacteria, rodents, weeds, nematodes, and other pests that damage crops. Thus, the use of pesticides during cultivation is of great importance for the quality of the harvest and yields. However, pesticides and the products generated during their degradation maybe dangerous for different environmental compartments (e.g. surface and groundwater). One of the major challenges for environmental conservation is the contamination of water resources by pesticides and other micropollutants (anthropogenic chemicals like pharmaceuticals, drugs, etc.), which are constantly released^3–5^. Therefore, there is a need for research on the concentration, fate, and behaviour of pesticide residues in the environment. Some pesticides are highly mobile and therefore, can easily migrate to the soil, water, and air, so the potential risk arises^6^. The aquatic environment may be contaminated with pesticides because of runoff, agricultural storm-water discharges, and return flow from irrigated fields^7–9^. The drainage systems facilitate the migration of agricultural contaminants, including pesticides, through rapid and direct transport from the soil zone and shallow groundwater to surface water^10^. Polar pesticides are more soluble and migrate faster to the surface and groundwater, while non-polar pesticides accumulate in the bottom sediments and suspension. Only the most persistent and mobile pesticides can migrate into the groundwater, and most of these compounds are absorbed in the upper soil layer^11^. However, pesticides have been detected in surface water, groundwater, and drinking water^12–17^. The use of contaminated water can adversely affect human health^5,18^. Consumption of water contaminated with pesticides may result in various health hazards, such as cancer and neurological and reproductive disorders^6,19,20^.

Due to the negative impact of pesticides on human health, the Drinking Water Directive 98/83/EC^21^ sets a maximum concentration of 0.1 mg/L for each individual pesticide and their degradation products and 0.5 mg/L for the sum of all pesticides present in a sample. Moreover, in the new EU directive 2020/2184^22^ on the quality of water intended for human consumption, risk based approach of pesticide monitoring is recommended for the identification of pesticides that are likely to be present in a given environment.

The occurrence of pesticides in drinking water owing to surface water intake has been reported in previous studies^14,23,24^. Riverbank filtration (RBF) is a common method of water exploitation, where the exploited water quality is highly dependent on surface (source) water quality. Well fields that use RBF are also exposed to pesticide pollution^25–27^. The specificity of the RBF well fields is that the wells are supplying with surface water originating from rivers or lakes. The wells are located at a small distance and cause water to flow from watercourses or reservoirs^28,29^. During underground water passage through the aquifer media biological, chemical, and physical processes such as biodegradation, adsorption, and chemical precipitation occur, which results in a natural improvement in water quality^29,30^. However, this is not always adequate to remove all contaminants, particularly organic micropollutants. Depending on the distance and travel time, the contaminants are removed to a greater or lesser extent^31–33^. Pesticides, like other organic micropollutants, migrate from rivers to wells^34,35^. The cumulative effect of chronic exposure to pesticides may be harmful to human health^23,24^. Therefore, a health risk assessment of contaminated water ingestion must be conducted.

Pollution of surface water with pesticides generally exhibits seasonal variation. An increase in water pollution can be observed during periods of intensive use of pesticides^24,36^. In addition to the increase in concentrations during the period of pesticide use, a change in the types of detected substances can be observed during the annual cycle.

The aims of this research were (i) to analyse the seasonal variation of pesticides and the behaviour of individual pesticides in the river and RBF water, and (ii) to assess the potential health risks.

## Material and methods

### Site description

**Figure 1:**
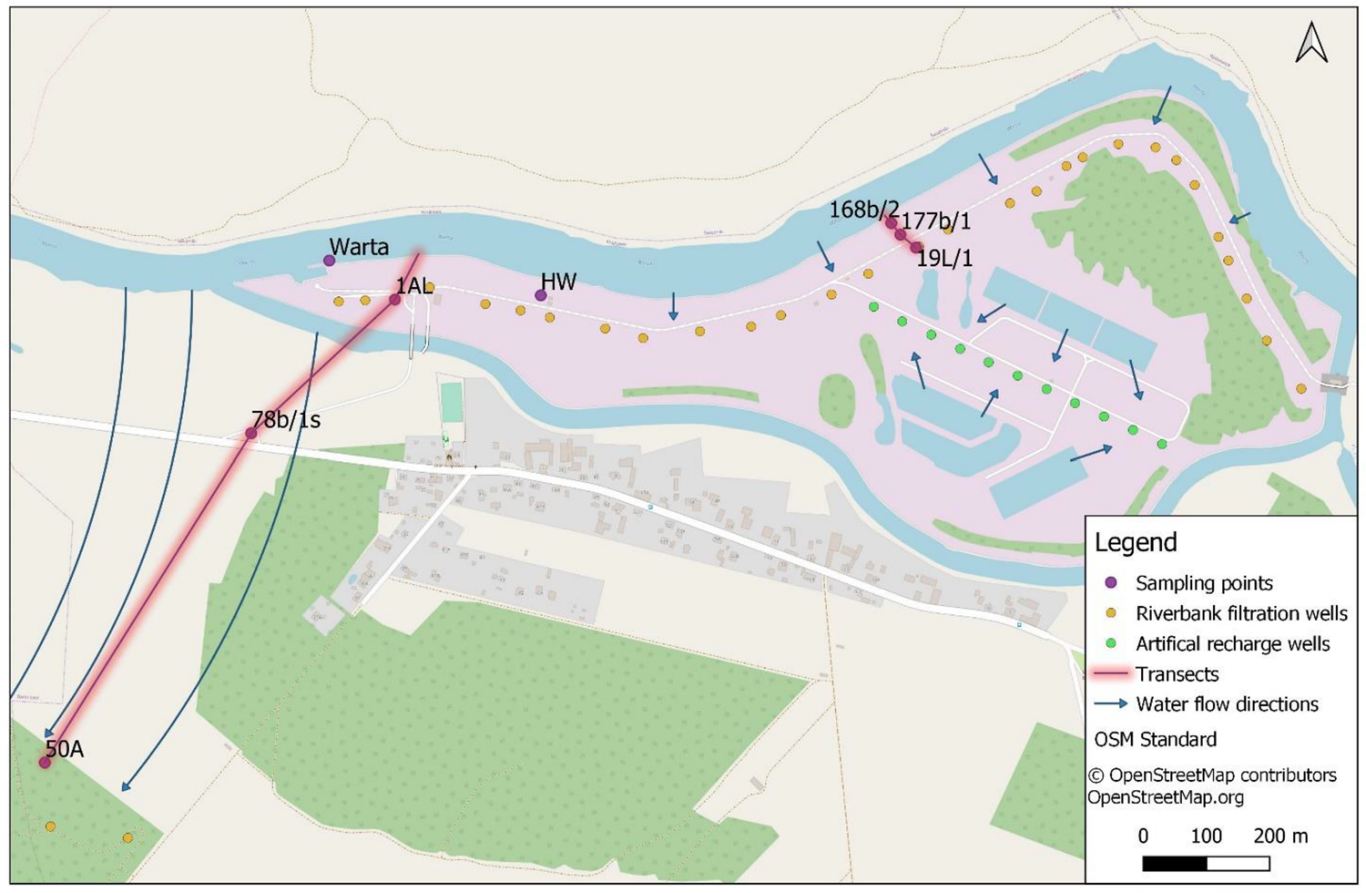
Map of the study area.

The research was conducted at the Mosina-Krajkowo RBF well field (Fig. 1). The well field supplies drinking water to a large agglomeration (900,000 inhabitants) of the Poznań city in Poland. The selected site is located on the bank of the Warta River in Krajkowo. There are favourable hydrogeological conditions, because of the sediments of two groundwater bodies overlap, that is, the Warszawa-Berlin ice-marginal valley aquifer (shallow) and the Wielkopolska buried valley aquifer (deep). The total thickness of the water-bearing sediments is 40 m. The shallow aquifer is composed of coarse sands in the deeper section and fine sands in the upper section, and the deep aquifer comprises coarse sands and gravels beneath the fine sands. These aquifers are locally separated by a glacial till^37^. The well field is located near the forest and agricultural areas. It consists of 56 vertical wells located on the higher terrace, 400–1000 m away from the river; 29 vertical wells located in the flood plain, 60–80 m from the riverbank; 11 vertical wells supplied by artificial ponds; and the horizontal well (HW).

### Sampling and sample analysis

The study was conducted during the annual cycle (from August 2017 to August 2018). Samples were collected in seven sampling campaigns (August and November 2017; February, May, June, July, and August 2018). The increased frequency of research during May–August 2018 was because of the intensive use of pesticides in agriculture during this time of the year. The data from the first three of the seven sampling campaigns were previously published^15,27^.

Samples were collected at eight sampling points: one in the river, two at observation wells, and five at production wells: HW and four vertical wells (Fig. 1). The sampling points were selected to represent different types of wells and different distances from the river (Table 1). Each production well was continuously pumped before sampling, and the observation wells were pumped using a portable pump (MP-1 type, Grundfos). The water in the well column was flushed a minimum of three times before sampling. Water was sampled in 1000 mL high-density polyethylene (HDPE) bottles. The bottles were filled to prevent degassing. The water samples were transported to the laboratory of the Institute of Plant Protection, National Research Institute in Poznań (Department of Pesticide Residue Research) on the same day for analysis.

**Table 1:** Sampling points characterisation.

Sampling point	Location	Distance from the riverbank (m)	Depth of the well screen (m)
Warta River	–	–	–
Horizontal well	Drains under the river bottom	–	5 m below the river bottom
168b/2	Flood plain	11	5.5–7.5
177b/1	Flood plain	38	12.5–14.5
19L	Flood plain	64	24.0–32.0
1AL	Flood plain	82	16.5–32.5
78b/1s	Higher terrace	250	18.0–28.0
50A	Higher terrace	680	31.8–41.8

Immediately after arrival at the laboratory, the samples were filtered, and 500 ml of water sample was taken for pesticide extraction. All selected pesticides were isolated from water samples by solid phase extraction (SPE, graphitised carbon black), followed by reverse-phase ultra-performance liquid chromatography with quadrupole mass detection (RP-UPLC-MS/MS)^38,39^. The studies included more than 160 active substances in pesticidess from different pesticide activities. The identified pesticides during the annual cycle included herbicides, fungicides, and insecticides, and the quantification limits for concentrations of individual compounds were from 0.005 to 0.02 µg/L. All methods were carried out in accordance with relevant guidelines and regulations.

### Health risk assessment

Based on published standards an assessment of non-carcinogenic risk to human health was conducted for children (6 years old) and adults (70 years old) and the risks were calculated using hazard quotients (*HQ*s)^3,24,36^ The *HQ* values were estimated using Eq. (1) with the assumption that the river water and RBF water were untreated. An *HQ* value > 1 indicates that significant risk could occur, and *HQ* < 1 indicates no significant risk^3,24^.

1 $$HQ=\frac{CDI}{ADI}$$

where, *CDI* is the protracted daily intake of pesticides through ingestion (mg/kg/day) estimated using Eq. (2), and *ADI* is the acceptable daily intake (mg/kg/day)^3,40^. The *ADI* values come from and are approved by the United States Environmental Protection Agency (US EPA) and Food and Agriculture Organization (FAO) of the United Nations reports.

2 $$CDI=\frac{C\ x\ IR\ x\ EF\ x\ ED}{BW\ x\ AT}$$

where, *C* is the concentration of a pesticide in a river or RBF water (mg/L), *IR* is the water ingestion rate (0.87 L/day for a 6 year-old child, 1.41 L/day for a 70-year-old an adult), *EF* is the exposure frequency (365 days/year), *ED* is the exposure duration (6 years for a child, 70 years for an adult), *BW* is the body weight (20 kg for a child and 70 kg for an adult), and *AT* is the average time (lifespan) (2190 days for child and 25,550 days for an adult) (Table 2)^3,24^.

**Table 2:** Parameters for hazard quotient (HQ) calculations^3,41^.

Parameter	6 year old child	70 year old adult
Water ingestion rate (IR) (L/day)	0.87	1.41
Exposure frequency (EF) (days/year)	365	365
Exposure duration (ED) (years)	6	70
Body weight (BW) (kg)	20	70
Average time (AT) (days)	2190	25,550

## Results

### Occurrence of pesticides in surface and riverbank filtration (RBF) water samples

Among the 164 analysed pesticides, 22 were detected (LOQ > 0.005 μg/L) in the Warta River (lotic surface water) with pesticide concentrations ranging 0.031–0.472 μg/L. Generally, the concentrations in the Warta River were higher than those at the other sampling points (Table 3, Supplementary file). The exception was in June 2018, when a higher concentration of the sum of pesticides (0.197 μg/L) was observed in the observation well, 168b/2, than that in the Warta River (0.111 μg/L).

**Table 3:** Total concentrations of pesticides at each sampling point in all sampling campaigns, *nd* not detected.

Date	Unit	Warta	HW	168b/2	177b/1	19L	1AL	78b/1 s	50A
August 2017	μg/L	0.112	0.086	0.112	0.068	0.049	0.045	0.019	nd
November 2017		0.171	0.137	0.058	0.076	0.058	0.064	0.023	nd
February 2018		0.031	0.024	0.005	nd	0.014	0.014	0.024	0.008
May 2018		0.472	0.191	0.114	0.027	0.045	0.042	0.034	0.006
June 2018		0.111	0.096	0.197	0.129	0.052	0.055	0.018	nd
July 2018		0.148	0.087	0.064	0.059	0.058	0.041	0.018	nd
August 2018		0.100	0.035	0.035	0.035	0.023	0.022	0.009	nd

However, in the case of RBF water samples, 21 active substances were detected. Pesticides were detected at each sampling point at various concentrations (Table 3, Supplementary file). The highest concentrations and the lowest reduction in pesticides were observed in 168b/2 (maximum 0.197 μg/L) and HW (0.191 μg/L) (two points located nearest to the river). However, at each subsequent point, the concentrations decreased compared to that in the river water. Similar concentrations were detected in the two analysed wells located in the flood plain during each sampling campaign. Lower concentrations occurred in 78b/1 s, whereas no pesticides or very low concentrations were detected in the farthest sampling point (50A) located at the higher terrace. The concentration reduction increased as the distance between the river and well increased. Notably, only persistent pesticides were detected at the most distant points, for example, the herbicides chlorotoluron and isoproturon^42^.

**Figure 2:**
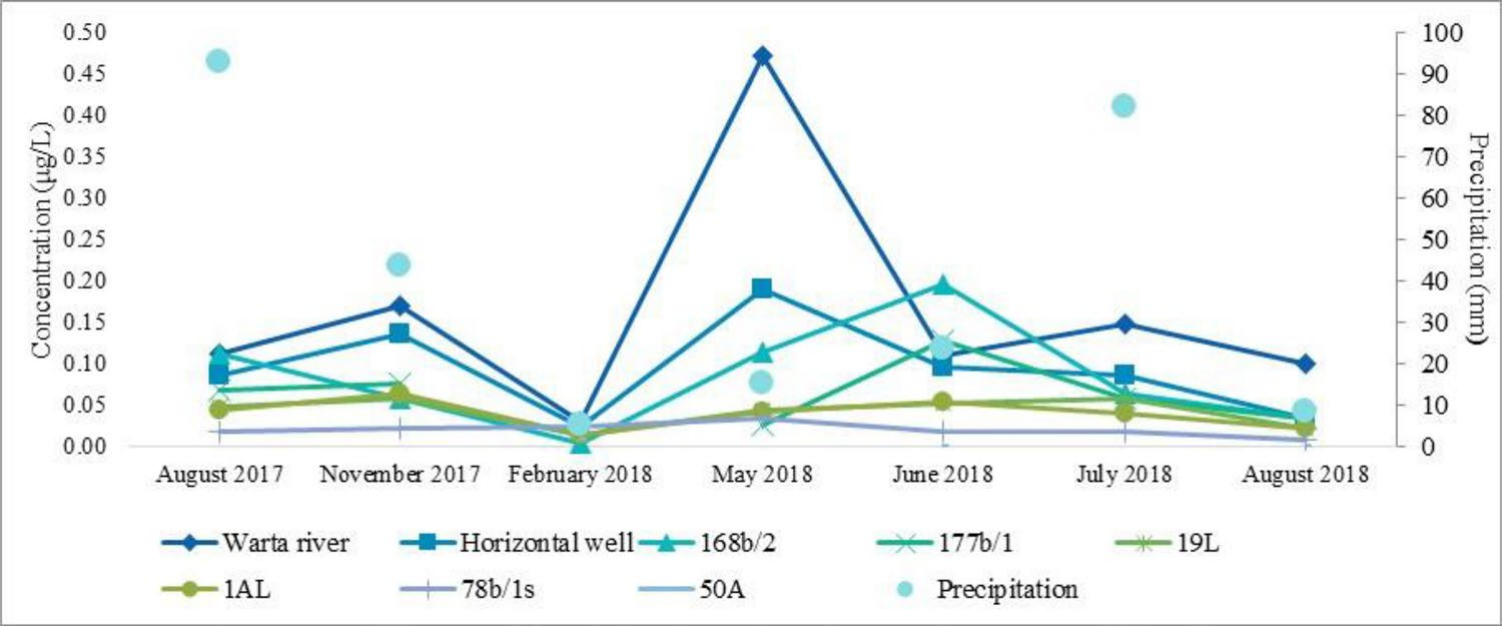
Total concentrations of pesticides in the Warta River, wells, and observation wells in each sampling campaign, and total monthly precipitation.

The concentrations of pesticides in the surface and groundwater changed with the study period (Fig. 2). The highest pesticide concentration in surface water occurred in May 2018 (0.472 μg/L). The lowest concentrations were recorded in February (minimum 0.031 μg/L). In the remaining months, the concentrations of pesticides in river water were not variable (ranging 0.100–0.171 μg/L). The situation was slightly different for RBF water. The highest pesticide concentrations were observed in June 2018 (Fig. 2). Similar to the Warta River, the lowest concentrations occurred in February 2018. Generally, the concentration of pesticides was lowest in the winter months. Significantly lower concentrations of pesticides in RBF water were observed in August 2018 (0.009–0.035 μg/L) than in August 2017 (0.019–0.112 μg/L). This may be due to the lower rainfall that occurred in July 2018 (monthly total of 10 mm) than in July 2017 (monthly total of 93 mm) (Fig. 2).

In August 2018, fewer compounds (8 pesticides) were found in water than in August 2017 (13 pesticides). Substances that occurred in August 2017 and 2018, had lower concentrations in 2018. The same compounds appeared at lower concentrations in August 2018. Concentrations in the Warta River were similar, at 0.112 µg/L in August 2017 and 0.100 µg/L in August 2018. In 2018, pesticides in the 168b/1 observation well were significantly reduced (from 0.112 µg/L in August 2017 to 0.035 µg/L in August 2018). No pesticides were detected in the 50A well in both sampling campaigns.

### Seasonal variation of pesticides in water samples

**Figure 3:**
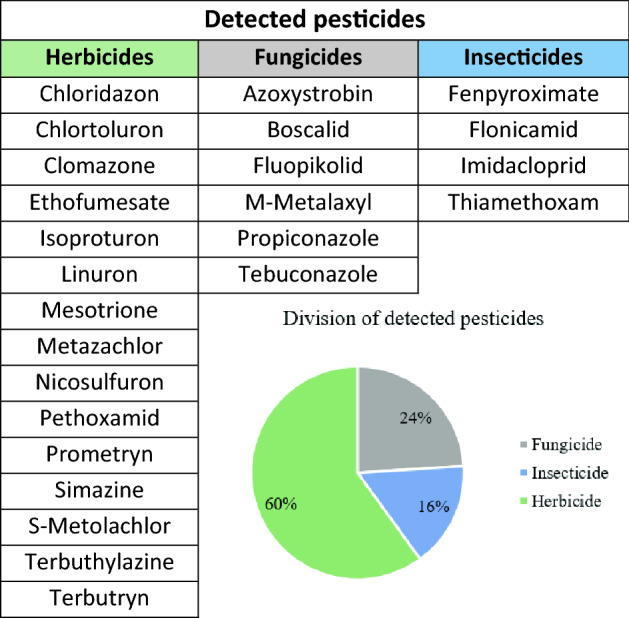
Detected pesticides divided into three groups: herbicides, insecticides, and fungicides.

Among the 25 substances detected in the water samples, 15 were herbicides, 6 fungicides, and 4 insecticides (Fig. 3, Table 4). Approximately 60% of the 25 detected substances were herbicides, possibly due to their popularity and ease of application in plant protection, that is, through soil spraying and spraying plants during the early stages of vegetation. Insecticides are less common in the analysed aquatic environments.

**Table 4:** Pesticides in river and RBF water.

Month	Number of detected substances		Sum of concentrations		Pesticides detected in the highest concentrations	
	Warta River	RBF water	Warta River (µg/L)	RBF water (µg/L)	Warta River (µg/L)	RBF water (µg/L)
August 2017	10	12	0.112	0–0.112	Metazachlor (0.036) Azoxystrobin (0.012) Nicosulfuron (0.012)	Metazachlor (0.015) Nicosulfuron (0.012–0.03) Chlorotoluron (0.007–0.018)
November 2017	12	12	0.171	0–0.137	Chlorotoluron (0.047) Metazachlor (0.021) Nicosulfuron (0.019)	Chlorotoluron (0.005–0.032) Nicosulfuron (0.017–0.024) Izoproturon (0.007–0.018)
February 2018	4	3	0.031	0–0.024	Chlorotoluron (0.012)	Chlorotoluron (0–0.013) Isoproturon (0–0.015)
May 2018	15	9	0.472	0.006–0.191	Nicosulfuron (0.065) Ethofumesate (0.058) S-metolachlor (0.055)	Nicosulfuron (0.015–0.05) Imidacloprid (0–0.043)
June 2018	11	15	0.111	0–0.196	Nicosulfuron (0.045)	Nicosulfuron (0.03–0.088)
July 2018	9	10	0.148	0–0.087	Nicosulfuron (0.057)	Nicosulfuron (0.012–0.036)
August 2018	7	6	0.100	0–0.035	Nicosulfuron (0.057)	Imidacloprid (0.007–0.017) Chlorotoluron (0.005–0.012)

**Figure 4:**
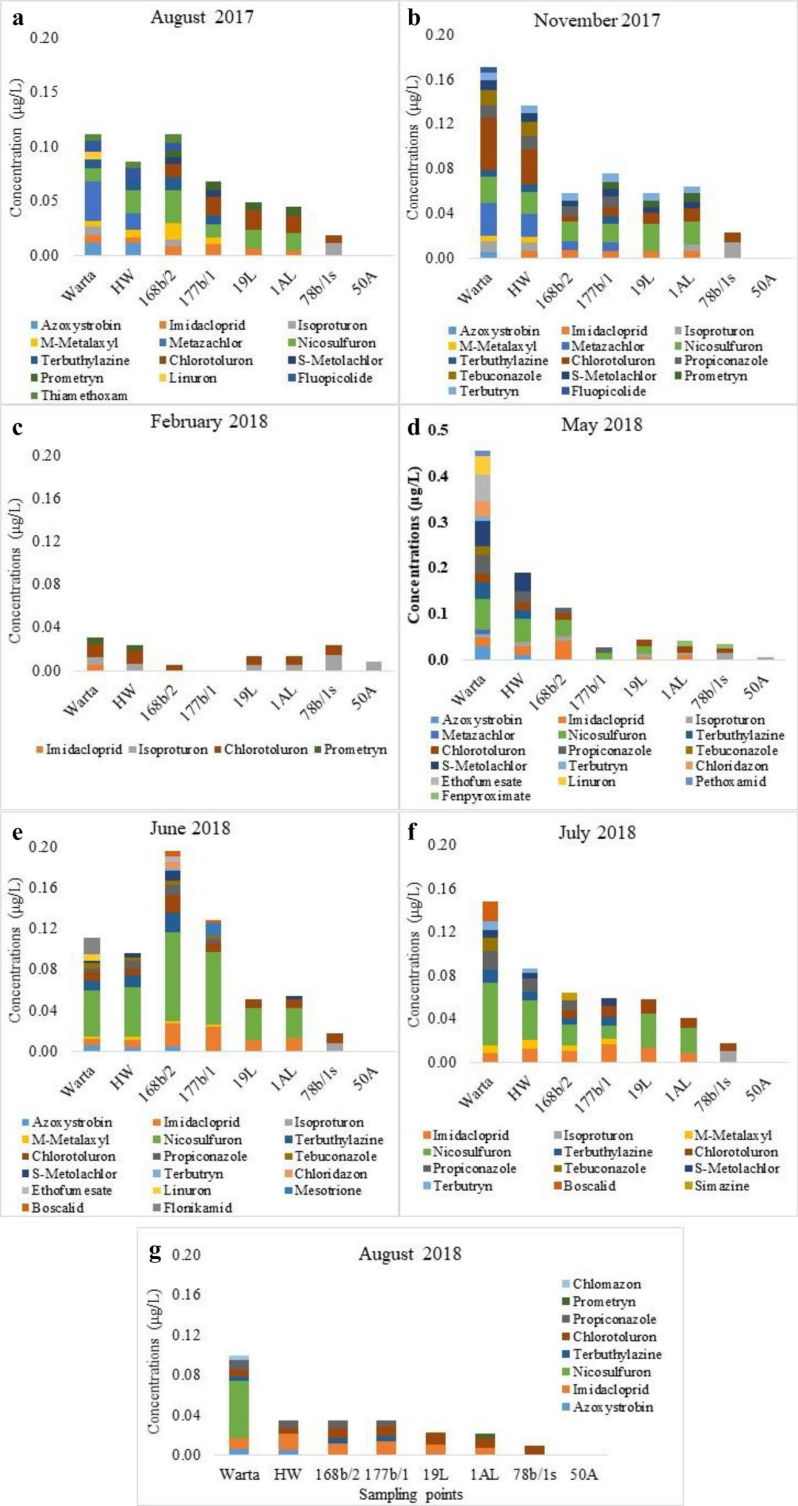
Total concentrations of pesticides at each sampling point divided into individual compounds in (**a**) August 2017, (**b**) November 2017, (**c**) February 2018, (**d**) May 2018, (**e**) June 2018, (**f**) July 2018, and (**g**) August 2018.

The highest number of pesticides in surface water was detected in May 2018 (15 substances), whereas in RBF water in June 2018 (15 substances) (Table 4, Fig. 4). The lowest number of substances both in surface and RBF water was detected in February 2018. Similarly, it was in case of concentration. The highest sum of concentration in surface water was detected in May 2018 (0.472 µg/L) and in RBF water in June 2018 (max. 0.191 µg/L). In both surface and RBF water, the lowest sum of pesticide concentrations was detected in February 2018. In the Warta River it was 0.031 µg/L and in RBF water 0–0.024 µg/L, respectively). The most frequently detected pesticide in the highest concentration both in surface water and RBF water was nicosulfuron. Chlorotoluron also often appeared in RBF water in high concentrations.

Generally, in the Warta River and at the closest points to the river, primarily the same substances were present. Most of the same substances occurring in the river also occurred in the HW. However, a similar trend was observed in the observation wells (168b/2 and 177b/1). At the other sampling points, the number of substances present was significantly reduced. Imidacloprid, nicosulfuron, isoproturon, and chlorotoluron were dominant in the drinking water wells (19L and 1AL). Notably, at the points further from the river, particularly 78b/1s and 50A, some substances were detected which did not occur in the river or at HW during the sampling campaigns in the given series, for example, isoproturon and chlorotoluron. Moreover, priority substances in the field of water policy not allowed for use in today's agriculture were detected in water samples; prometryn and simazine were banned within the European Union in 2007, and isoproturon in 2017.

**Figure 5:**
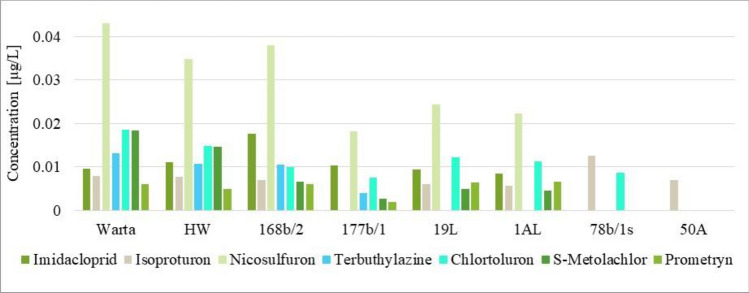
The total concentrations of individual pesticides exhibiting the highest concentrations that occurred at a given sampling point.

Seven pesticides occurring in the surface and RBF water had the highest concentrations: imidacloprid, isoproturon, nicosulfuron, terbuthylazine, chlorotoluron, S-metalachlor, and prometryn (Fig. 5). Among these compounds, nicosulfuron stands out the most in the figure, exhibiting the highest concentration at six sampling points.

### Health risk assessment

The non-carcinogenic human risks for a single pesticide posed by the ingestion of surface and RBF water were calculated as HQs. The HQ values are summarised in Table 5. HQs were calculated for azoxystrobin, boscalid, fluopicolide, fenpyroximate, propiconazole, tebuconazole, imidacloprid, thiamethoxam, and mesotrione, owing to the availability of acceptable daily intake values. The values were similar and ranged from 1E−05 to 1E−07. In general, the highest values were obtained for tebuconazole (1E−05) and the lowest for propiconazole (1E−07). The risk for children was higher than that for adults.

**Table 5:** HQ values indicating non-carcinogenic human risks.

Pesticide	Sampling point	ADI (mg/kg/day)	C (mg/L)		HQ (child)		HQ (adult)	
			Min	Max	Min	Max	Min	Max
Azoxystrobin	Warta	0.2^43^	5.50E−06	3.00E−05	6.53E−06	1.20E−06	3.26E−05	6.04E−07
	HW		4.00E−06	1.20E−05	8.70E−07	2.61E−06	4.03E−07	1.31E−05
Boscalid	168b/2	0.04^44^	5.50E−06		5.98E−06		2.77E−06	
	177b/1		3.50E−06		3.81E−06		1.76E−06	
Fluopikolid	Warta	0.08^45^	5.00E−06	1.10E−05	2.72E−06	5.98E−06	1.26E−06	2.77E−06
	HW		1.20E−05		6.53E−06		3.02E−06	
	168b/2		8.00E−06		4.35E−06		2.01E−06	
Fenpyroximate	1AL	0.01^46^	1.20E−05		5.22E−05		2.42E−05	
	78b/1 s		9.00E−06		3.92E−05		1.81E−05	
Propiconazole	Warta	0.7^47^	1.00E−05	3.90E−05	6.21E−07	2.42E−06	2.88E−07	1.12E−06
	HW		8.00E−06	2.40E−05	4.97E−07	1.49E−06	2.3E−07	6.91E−07
	168b/2		8.00E−06	1.20E−05	4.97E−07	7.46E−07	2.3E−07	3.45E−07
	177b/1		5.00E−06	1.20E−05	3.11E−07	7.46E−07	1.44E−07	3.45E−07
Tebuconazole	Warta	0.03^48^	5.00E−06	2.00E−05	7.25E−06	2.90E−05	3.36E−06	1.34E−05
	HW		2.50E−06	1.30E−05	3.63E−06	1.89E−05	1.68E−06	8.73E−06
	168b/2		4.50E−06		6.53E−06		3.02E−06	
	177b/1		3.00E−06		4.35E−06		2.01E−06	
Imidacloprid	Warta	0.06^49^	5.00E−06	1.90E−05	3.63E−06	1.38E−05	1.68E−06	6.38E−06
	HW		5.00E−06	1.80E−05	3.63E−06	1.31E−05	1.68E−06	6.04E−06
	168b/2		8.00E−06	4.30E−05	5.80E−06	3.12E−05	2.69E−06	1.44E−05
	177b/1		7.00E−06	2.40E−05	5.08E−06	1.74E−05	2.35E−06	8.06E−06
	19L		6.00E−06	1.40E−05	4.35E−06	1.02E−05	2.01E−06	4.7E−06
	1AL		5.00E−06	1.30E−05	3.63E−06	9.43E−06	1.68E−06	4.36E−06
Thiamethoxam	Warta	0.08^48^	6.00E−06		3.26E−06		1.51E−06	
	HW		6.00E−06		3.26E−06		1.51E−06	
	168b/2		8.00E−06		4.35E−06		2.01E−06	
Mesotrione	177b/1	0.5^44^	1.20E−05		1.04E−06		4.83E−07	

*ADI* acceptable daily intake; *C* the concentration of a pesticide in a river or RBF water, *HQ* hazard quotients.

Based on The Hazardous Substances Data Bank (HSDB) the carcinogenic risk was also assessed^50^. Propiconazol, tebuconazole are possible human carcinogenic. Azoxystrobin and mesotrione are classified as not likely to be carcinogenic to humans. Boscalid has suggestive evidence of carcinogenicity, but is not sufficient to assess humans carcinogenic potential. No evidence for carcinogenicity was observed in rats administered fluopicolide in food for 24 months. Imidacloprid and thiamethoxam are non-carcinogenicity for humans.

## Discussion

Water pollution caused by pesticides is notably high in the RBF well field, where the wells are supplied by the infiltration of surface water^25–27^. Thus far, previous studies have proven that the highest concentration of pesticides occurs in the surface water, and the contaminants decrease along the flow path to the wells. Research conducted on the Mosina-Krajkowo RBF well field has shown a high but incomplete reduction of contaminants^15,27,33^. Overall, a continuation of the aforementioned research in this study confirmed these findings. The concentrations of pesticides were reduced with increasing distance from the river. The highest concentrations were observed at surface water and HW. This trend changed in June 2018, when higher concentrations were observed in the observation wells. This could be due to the very high concentrations of pesticides in the river in the previous month (May 2018). Polluted water was supplied to wells in June 2018. The relationship between pesticide concentration and rainfall was also revealed, primarily in August 2017 and 2018. Previous research has focused on the occurrence and reduction of pesticides as a group of pollutants^27^. This study focused on the individual substances; pesticides were not considered as a group, but the behaviours of individual substances were analysed.

The study was conducted during the annual cycle and indicated the period of the year when water was most susceptible to pollution. The highest concentration of pesticidess in surface water could be combined with the period of increased chemical crop protection. Farmlands are often located close to rivers, which causes pesticides to rapidly migrate to the surface water^51,52^. In the study area, plant protection is conducted mainly between March and October, with particularly high protection measures adopted during spring. This was confirmed by the high concentration of pesticides in May and June. The lowest concentrations of pesticides in February could be related to the end of the plant-protection period. This was consistent with the conclusions of the research by Battaglin et al.^53^ conducted in US streams, where the occurrence of fungicides was associated with their use in drainage basins. In the aforementioned studies, the dominant periods were late summer and early autumn. The difference in periods may result from other climate conditions and the cultivation of other plants. Notably, the present study included RBF water, where pollution is detected after a long time compared to surface waters. The delay is due to the specificity of the RBF well field. River water supplying the wells infiltrates from the river into the aquifer, which lasts from a few days to several weeks^33^.

The highest concentrations of pesticides in both river and RBF water were in spring. However, varied concentrations were not the only differences. Individual substances also occurred seasonally. Depending on the sampling campaign, other substances were detected and reached the highest concentrations at the selected sampling points. It could be associated with the period of use and the persistence of the considered substances. In June and July 2018, nicosulfuron was detected at the highest concentrations. It was also found in other months (August and November 2017, May and August 2018), but at lower levels. Similar behaviour was observed for imidacloprid. It occurred at higher concentrations in June, July, and August 2018, and at lower concentrations in August, November, and May 2017. The same findings were obtained in the studies carried out in the Cachapoal River basin in Central Chile, where some of the pesticides were present only in the winter season (terbuthylazine, simazine, atrazine, and DIA), and some only at the end of the summer season (chlorpyrifos, and DEA)^54^.

During the spring and summer months, more substances were detected. This was particularly significant in river water. In February, two substances were most frequently present in the samples. Chlorotoluron and isoproturon occurred both in the river and in wells (even those farthest from the river). These two substances were also present in the other sampling campaigns, particularly at the farthest points (78b/1s, 50A).

The most common substances used for plant protection in this research area were herbicides. This is consistent with the worldwide consumption (the amount used in mass units) of pesticides, at 47.5% herbicides, 29.5% insecticides, and 17.5% fungicides^52^. Herbicides are also prevalent in other European countries, for example, Greece^36^. Herbicides are pesticides used to control weeds in crops. The most common pesticidess present in the highest concentration in collected samples was a sulfonylurea herbicide, nicosulfuron, which is widely used for weed control in corn^55^. The next was chlorotoluron, a phenylurea herbicide used to control grass weeds in cereal or fruit production. Chlorotoluron is also used to protect crops, primarily during late autumn and early spring. Prolonged use of chlorotoluron may cause its accumulation in the environment^56^. S-metalachlor is commonly used as a herbicide for selective weed control^57^. The obtained results are in contrast with the findings of the study conducted in the Choluteca River Basin of Honduras, where the pesticides present in water are weakly correlated with the current pesticide applications^58^.

Although insecticides are the least frequently occurring group of pesticides, imidacloprid was observed to have high concentrations. These results are of concern because imidacloprid is one of the most toxic insecticides for bees^59^. In addition, neonicotinoid insecticides are toxic to aquatic invertebrates^60,61^. This toxic and persistent neonicotinoid insecticide is also observed at high concentrations in the area of intense corn and soybean production in the Midwestern United States^62^ and agricultural regions of southern Ontario in Canada^63^. Sultana et al.^63^ suggested further research to determine whether imidacloprid pollution is a global problem. The literature review and research conducted have proven this. Additionally, banned pesticides (isoproturon, prometryn, and simazine) were detected in the surface and RBF water. However high stability and durability, as well as low concentrations of these substances suggest that these is residues from past use.

Pesticides are toxic and persist in the environment. Some of them are not reduced or only partially eliminated, during the natural processes (RBF) and through treatment systems^64,65^. Water from the RBF well field is used for consumption; therefore, risk assessment studies are necessary. None of the HQs (for children or adults) for a single pesticide exceeded one, which implied that no significant health risk occurred due to the potential daily ingestion of river or RBF water. Similar results have been achieved in research on rivers in northern Greece, wherein the potential non-carcinogenic risk is low^36^. Research results from Japan indicate that diazinon and fenitrothion pose a high risk^3^. In the study conducted on the Mosina-Krajkowo well field, these substances were not present. Research conducted in drinking water in Ethiopia showed that there was no acute risk; however, chronic human health risks were observed^23^. The occurrence of hazards in different locations necessitates the constant monitoring of water quality and potential risk assessment. Equally important is research into the cumulative health risk of consuming water containing a mixture of pesticides. However, standards are needed to provide for the interaction between pesticides.

## Conclusions

Research conducted in the annual cycle showed the presence of pesticides in the Warta River and RBF well field water, where the river is the main source of water. Of the 164 analysed pesticides, 25 were detected at the sampling points. Overall, the highest concentrations were observed in the Warta River. The concentrations in the wells were lower, which was the effect of the processes that occurred during riverbank filtration (RBF). Water pollution caused by pesticides shows seasonal variation. The highest pesticide concentrations occurred during the period of using pesticides in agriculture.

The pesticides found in collected water samples included herbicides, insecticides, and fungicides. Approximately 60% of the detected substances were herbicides, because of their popularity and spraying techniques. The occurrence of individual pesticides was also analysed. The greatest variation in pesticides, similar to the highest concentrations, was observed in the period of increased pesticide use by farmers. In the winter month (February 2018), only the most persistent pesticides were detected (chlorotoluron, imidacloprid, isoproturon, and prometryn). Most pesticides were detected in June 2018 (17), May 2018 (15) and November 2017. At the highest concentrations in surface and RBF water, seven pesticides occurred: imidacloprid, isoproturon, nicosulfuron, terbuthylazine, chlorotoluron, S-metalachlor, and prometryn. Nicosulfuron, chlorotoluron, and S-metalachlor are used for weed control, for example, in corn or cereal, which are common in the study area. Isoproturon, prometryn, and simazine have been banned in the European Union.

During the research, a very toxic substance, imidacloprid, was detected at high concentrations. This result confirmed that this insecticide is a global problem. Literature data from different parts of the world describe the presence of this substance at high concentrations.

The non-carcinogenic human risks posed by ingestion of surface and RBF water were calculated as the HQs for a single substances: azoxystrobin, boscalid, fluopicolide, fenpyroximate, propiconazole, tebuconazole, imidacloprid, thiamethoxam, and mesotrione. These values did not exceed one, which indicated a low risk. Tebuconazole exhibited the highest values of HQs, and propiconazole had the lowest. The potential risk for children was slightly higher than that for adults. The risks calculated for individual substances for surface and RBF water were similar; however, both were low. A further step should be to identify the risk of multi-pesticide consumption and calculate the cumulative risk.

Conducted research performed for RBF site, where groundwater quality is strongly depending on surface (source) water quality, covering the entire year allowed to show the variability of pesticide concentrations in the river and wells in relation to the periods of their use in agriculture and the hydrological situation (rainfall). It was also determined to what extend various pesticides are removed during underground flow of water from the river to wells. The presence of banned pesticides has been also identified. The presented research indicate the importance of pesticide monitoring at RBF sites and can be used to determine the principles of pesticides monitoring and the needs for further engineering water treatment in order to protect public health.

## Supplementary Information

Supplementary Information.
